# A normative microbiome is not restored following kidney transplantation

**DOI:** 10.1042/CS20230779

**Published:** 2023-10-17

**Authors:** Hannah Craven, Helen Erlandsson, Dagmara McGuinness, David H. McGuinness, Denise Mafra, Umer Zeeshan Ijaz, Peter Bergman, Paul G. Shiels, Peter Stenvinkel

**Affiliations:** 1 University of Glasgow, College of Medical, Veterinary and Life Sciences, School of Molecular Biosciences, Davidson Bld, Glasgow, U.K.; 2 Department of Clinical Science, Intervention and Technology, Division of Renal Medicine, Karolinska Institutet, Stockholm, Sweden; 3 School of Medicine University of Glasgow, U.K.; 4 Fluminense Federal University (UFF), Niterói, RJ, Brazil; 5 School of Engineering University of Glasgow, U.K.; 6 Department of Laboratory Medicine, Division of Clinical Immunology, Karolinska Institutet, Stockholm, Sweden; 7 Department of Clinical immunology and Transfusion Medicine, Karolinska University Hospital, Stockholm, Sweden

**Keywords:** chronic kidney disease, kidney transplantation, microbiome

## Abstract

Dialysis and kidney transplantation (Ktx) mitigate some of the physiological deficits in chronic kidney disease (CKD), but it remains to be determined if these mitigate microbial dysbiosis and the production of inflammatory microbial metabolites, which contribute significantly to the uraemic phenotype. We have investigated bacterial DNA signatures present in the circulation of CKD patients and those receiving a KTx. Our data are consistent with increasing dysbiosis as CKD progresses, with an accompanying increase in trimethylamine (TMA) producing pathobionts *Pseudomonas* and *Bacillus*. Notably, KTx patients displayed a significantly different microbiota compared with CKD5 patients, which surprisingly included further increase in TMA producing Bacillus and loss of salutogenic Lactobacilli. Only two genera (*Viellonella and Saccharimonidales*) showed significant differences in abundance following KTx that may reflect a reciprocal relationship between TMA producers and utilisers, which supersedes restoration of a normative microbiome. Our metadata analysis confirmed that TMA N-oxide (TMAO) along with one carbon metabolism had significant impact upon both inflammatory burden and the composition of the microbiome. This indicates that these metabolites are key to shaping the uraemic microbiome and might be exploited in the development of dietary intervention strategies to both mitigate the physiological deficits in CKD and enable the restoration of a more salutogenic microbiome.

## Introduction

Chronic kidney disease (CKD) is a component part of the ‘diseasome of ageing’, characterised by accelerated biological ageing, a low-grade inflammatory burden and an increased risk of vascular disease [1]. Significantly, microbial metabolites contribute to patients’ uraemic status and exacerbate the inflammatory burden [2]. Contributing to the inflammatory burden in CKD is the production of trimethylamine N-oxide (TMAO), which relies upon the conversion of dietary substrates, including choline and carnitine, from trimethylamine (TMA). This process requires enzymes only transcribed by members of the gut microbiota and once TMA has been produced and absorbed into the bloodstream, it is converted into TMAO by hepatic flavin monooxygenases (FMOs) [3]. TMAO then activates NF-κB mediated inflammatory processes and drives fibrosis in CKD.

Microbiotas are also capable of producing Nuclear factor erythroid 2-related factor 2 (Nrf2) agonists and activators from dietary substrates and can therefore contribute to the alleviation of oxidative stress in the ‘diseaseome of ageing’ [4]. Thus, a link exists between nutrition, reduced renal function and the gut microbiota; with it having the capacity to either exacerbate or alleviate inflammatory and oxidative burden, dependent on whether it is in a healthy or dysbiotic state. Such an assertion is in keeping with our previous findings in the general population, where accelerated biological ageing and diminished renal function associated with microbial dysbiosis and an imbalanced diet [5,6]. Kidney replacement therapies (KRT) with dialysis or kidney transplantion (KTx) remove uremic toxins and may decrease the uremic inflammatory burden [7]. While one would expect this to result in an improvement in physiological function, both treatment strategies can conversely aggravate the pro-inflammatory and pro-oxidative mechanisms present in patients, and adversely impact upon the ageing process [1]. To date, there has not been any study investigating whether KTx restores the gut microbiota.

We have previously employed an analysis of bacterial DNA signatures in the circulation as a marker for gut microbial changes where no faecal samples were available, investigating its role in the ageing process, renal function and how this is affected by exposome differences, principally nutrition and socioeconomic position [5]. Such an analytical approach is specifically pertinent to CKD, a condition in which microbial signatures are present in the circulation because of a leaky gut [8,9]. We employed this strategy to compare the circulatory bacterial DNA signatures of patients with CKD3-4, CKD5 initiating dialysis and patients undergoing living donor KTx (LD-KTx) at basal (i.e., immediately before surgery) and after one year of follow-up when the uremic milieu have normalised. Our hypothesis was that reversal of the uremic milieu following KTx would generate a more salutogenic (health promoting) micorbiome. Furthermore, we aimed to establish differences in the microbiota which may either improve or exacerbate the inflammatory burden that could be used as targets for intervention. The study was designed to identify factors driving the composition of the microbiota in CKD and to determine to what extent the uremic milieu drives dysbiotic changes.

## Methods

### Cohort details

Patients were recruited from ongoing cohort studies, conducted at Karolinska Institutet, Stockholm, Sweden. Ten CKD 3–4 patients, 10 incident dialysis patients (CKD5) and 50 patients undergoing LD-Ktx were included. All patients gave their written consent to participate in the study and there were no exclusion criteria. The studies were approved by the Swedish Ethical Review Authority in Stockholm (2008/1748-31/2 and 40/02). The study was conducted in adherence with the Helsinki and Istanbul declarations. Fasting blood samples were taken at baseline (i.e. before dialysis initiation or KTx surgery) and about one year after KTx. Blood analytes were measured as part of the clinical biochemistry service assocaited with the above studies at the Karolinska University Hospital, Stockholm, Sweden.

### DNA isolation and 16S amplicon library preparation for microbiome analysis

DNA was extracted from whole blood using QIAamp DNA blood maxi kit (Qiagen, Hilden, Germany) or Chemagen® magnet bead extraction kit (Revvity Chemagen Technologie GmbH, Baesweiler, Germany).16S libraries encompassing the V3-V4 regions were generated by Glasgow Polyomics as described in the supplementary materials. Whole blood samples from patients underwent 16S analyses to establish a snapshot of bacterial DNA signatures in the blood, resulting in the generation of 13481 ASVs in total.

### Bioinformatics and statistical analysis

#### Sequence Quality trimming and ASV Generation

Amplicon sequence variants (ASVs) were constructed, and statistical analyses as previously performed [10] and described in the supplementary materials.

## Results

### Microbial community diversity

**Figure 1:**
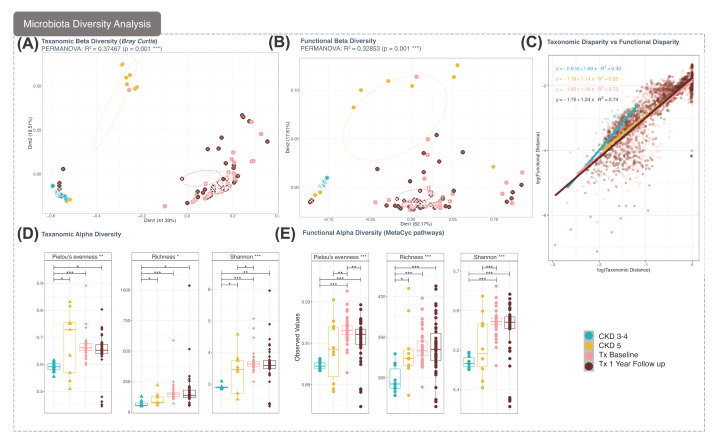
Microbial Diversity in terms of taxonomy and function (**A**) Beta diversity using Bray–Curtis distance measure and (**B**) hierarchical meta-storms (a hierarchical measure of functional beta diversity that takes into account redundancies in KEGG orthologies (KOs)and resolves beta diversity by collating abundances of KOs in a weighted fashion in terms of the pathways they belong to). PERMANOVA results for CKD groups (3–4 and 5) and transplant groups (Baseline and 1 year follow-up). For (a) and (b), the ellipses represent 95% confidence intervals of standard errors of each group. (**C**) A Linear regression model fitted to Log abundance of both paired wise distances (Bray-Curtis distances in *x*-axis and hierarchical meta-storm distances in *y*-axis). The slope of the fitted lines then reveals how functionally robust the communities are in terms of taxonomic perturbations. (**D,E**) Taxanomic and functional alpha diversity (Peilou’s evenness, Richness and Shannon entropy) for each group whereby lines connect two categories if the differences are significant (ANOVA) with *(*P*<0.05), **(*P*<0.01) or *** (*P*<0.001). Only samples with 5000 reads per sample have been included in these analyses.

Patient characteristics are described in Table 1. Analysis of beta diversity between samples, in terms of both taxonomy and functional capacity within the microbiome, indicated distinct microbiome composition for the respective CKD and KTx groups (*P*>0.001) (Figure 1A,B). Of note, four KTx follow-up samples clustered with the CKD3-4 group, and one sample of the KTx baseline group clustered with CKD5. We were unable to discern any reasonable distinctions of demography or clinical data from the available metadata. Our data also indicated an increasing dysbiotic state moving from CKD3–4 to CKD5. In particular, regression analysis (Figure 1C) indicated that at CKD3-4, the microbial ecosystem was volatile and small perturbations in a few community members would destabilise the functional potential of the microbial community. As such, the fitted regression line tilted more towards the *x*-axis suggesting that more perturbation in taxonomic composition would thus be required to give a substantial shift in overall microbial community function.

**Table 1:** Patient Characteristics

	CKD 3-4	CKD5	KTx	KTx 1 yr follow-up	*P*-value
	*N*=10	*N*=10	*N*=50	*N*=50	
Age, years	50 (36–63)	52 (43–61)	40 (29–55)	41 (30–56)	0.13
Male sex, *n* (%)	8 (80%)	8 (80%)	30 (60%)	30 (60%)	0.41
Diabetes mellitus, *n* (%)	1 (10%)	5 (50%)	5 (10%)	5 (10%)	0.005
CVD, *n* (%)	1 (10%)	2 (20%)	9 (18%)	NA	0.80
Malnutrition (SGA>1)	0 (0%)	0 (0%)	9 (19%)	NA	0.14
Weight, kg	78 (65–84)	81 (66–86)	65(60–76)	NA	0.013
BMI, kg/m^2^	23.5 (22.6-27.7)	25.1 (22.3-30.2)	23.0 (20.8-25.3)	NA	0.064
Systolic BP, mmHg	141 (122–153)	140 (131–155)	137 (126–152)	NA	0.81
Diastolic BP, mmHg	84 (76–89)	88 (82–96)	82 (72–88)	NA	0.30
Hand grip strength, %	109 (82–119)	86 (76–89)	92 (70–103)	NA	0.25
eGFR^a^ ml/min/1,73 m^2^	28 (19–40)	6 (4–7)	6 (5–8)	62 (45–74)	<0.001
Creatinine, μmol/L	226 (178–286)	737 (623–1,051)	702 (560–867)	119 (94–137)	<0.001
Total cholesterol, mmol/L	4.6 (4.2–5.4)	4.3 (3.7–4.9)	4.6 (4.2–5.2)	4.9 (4.1–6.2)	0.23
Triglyceride, mmol/L	1.7 (0.9–2.2)	1.35 (1.0–2.0)	1.6 (1.1–2.2)	1.5 (1.1–2.2)	0.89
Calcium, mmol/L	2.33 (2.23–2.35)	2.23 (2.05–2.39)	2.29 (2.23–2.46)	2.34 (2.30–2.45)	0.050
Phosphate, mmol/L	1.3 (1.1–1.6)	2.1 (2.0–2.4)	1.6 (1.3–2.0)	0.9 (0.8–1.1)	<0.001
Homocysteine, mmol/L	NA	NA	35 (25–45)	16 (14–22)	<0.001
hsCRP, mg/L	2.4 (0.8–8.5)	1.8 (0.9–9.1)	0.6 (0.3–2.1)	1.6 (0.5–4.2)	0.12
iPTH, ng/L	70 (42–118)	234 (148–272)	243 (176–439)	NA	<0.001
Eosinophil (10*9/L)	0.35 (0.20–0.55)	0.20 (0.16–0.22)	NA	0.10 (0.09–0.20)	<0.001
Basophil (10*9/L)	0.10 (0.10-0.10)	0.08 (0.04-0.10)	NA	0.09 (0.09–0.09)	<0.001
Beta-blocker	4 (40%)	8 (80%)	29 (58%)	23 (46%)	0.17
Calcium-blocker	4 (40%)	6 (60%)	22 (44%)	13 (26%)	0.12
ACEi/ARB	8 (80%)	10 (100%)	27 (54%)	43 (86%)	<0.001
Statin	1 (10%)	3 (30%)	18 (36%)	17 (34%)	0.45

a eGFR was measured using the CKD-EPI equation. ACEi/ARB; angiotensin-converting enzyme inhibitor/angiotensin receptor blocker.

**Figure 2:**
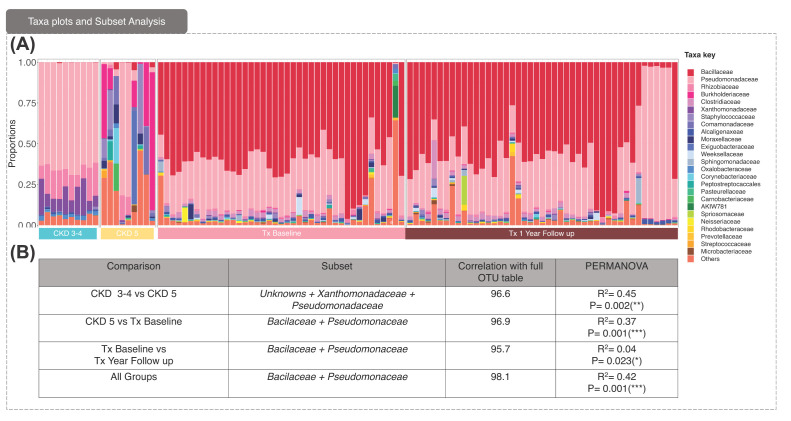
Taxa plots and subset analysis (**A**) Taxa plots representing the Top-25 most abundant families observed in all samples in the corresponding groups with the taxa key shown. (**B**) Subset analysis that implodes the abundance table down to a minimal subset of species that roughly explain the same beta diversity between samples as the full abundance table. The correlation value cut-off 0.95 was chosen and based on these subsets, the PERMANOVA values (*R*^2^ values explaining percentage variability between the groups) suggest them to be discriminatory.

Alpha diversity analysis showed that the KTx group was substantially distinct from CKD3-4/CKD5 (Figure 1D,E). The differences in beta diversity between groups is reflected in the taxa plot (Figure 2) showing the top 25 most abundant families present in each sample (ASV and genera level taxa plots are shown in Supplementary Figure S1). While the KTx groups were largely dominated by *Bacilaceae* and *Pseudomonaceae*, only *Pseudomonaceae* dominated the taxa profiles on CKD3-4 patients, while CKD5 patients differed again, with most patients in the group having distinct taxa profiles.

Subset analysis (Figure 2B), designed to determine any combination of subsets of ASVs which contributed significantly to microbiome changes, was performed to compare the cohorts (i.e., ‘CKD3-4 vs. CKD5’, CKD5 vs. KTx baseline, and ‘KTx baseline vs. follow up’). We also applied these analyses to compare all four groups (CKD3-4, CKD5, KTx baseline, KTx follow-up) in ‘all groups. *Xanthemodaceaea* and *Pseudomonaceae* and an unknown family were identified as contributing significantly (*P*=0.002) to shaping the microbiome in CKD3-4 and CKD5, with this subset contributing to 45% of the variation between these two untreated CKD groups. In KTx, *Bacilacea* and *Pseudomonaceae* were again identified as the families showing significant differences. Surprisingly, they had limited impact on the overall microbiome composition, only contributing to 4% of variation (*P*=0.023). These same families were also the subset that contributed to 37% variation (*P*=0.001) between the CKD5 vs. KTx Baseline groups, as well as 42% variation (*P*=0.001) between all four groups.

**Figure 3:**
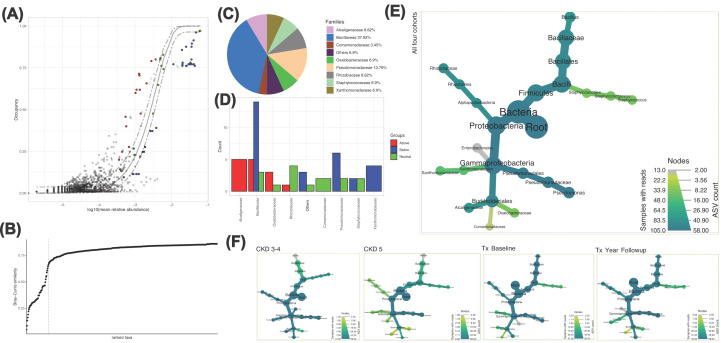
Core Microbiota (**A**) Core microbiome (red, green and blue points) identified through species occupancy abundance diagrams incorporating Site-Specific Occupancy (accounting for differences between CKD 3-4, CKD 5, KTx baseline, and KTx one year follow-up). (**B**) To identify the thresholds for core microbiome, we calculate the function C (that implicitly incorporates explanatory power of the chosen core subset in terms of capturing beta diversity). The dotted line represents ‘Last 2% decrease’ criteria where ASVs are incorporated in the core subset until there is no more than 2% decrease in beta diversity. (**C**) shows piechart of these ASVs resolved at Family level. Independently, a neutral model is fitted with those ASVs that fall within the 95% interval confidence intervals shown in green in (**A**), whilst non-neutral OTUs with observed frequency above the predicted frequency from the neutral model (selected by the host) are shown in red colours, and those with observed frequency below the predicted frequency from the neutral model (selected by dispersal limitation) are shown in green colours. (**D**) Then summarizes these OTUs at family level by giving their count. (**E**) Then shows the complete taxonomic coverage of these ASVs by collating abundances from all four cohorts together whilst (**F**) shows them separately for each cohort.

We also identified genera that remained unchanged in the *core microbiota* of a minimum of 85% of samples within a group, indicating that they are integral constituents of their respective microbiome (Figure 3). The CKD3-4 and CKD5 groups had similar *core* composition, with high abundance of *Pseudomonas* and *Staphylococcus*, both known producers of TMA [11]. Furthermore, *Staphylococcus* has been associated with lower levels of luminal H_2_S production [12], which might exacerbate cytotoxicity, given the cytoprotectant properties of H_2_S. The *core* community within the CKD5 group indicated that *Cutibacterium* was prevalent across samples in addition to *Staphylococcus* and *Pseudomonas*. Significantly, *Pseudomonas* was present in all four-*core microbiota*, indicating again its significance in CKD progression.

**Figure 4:**
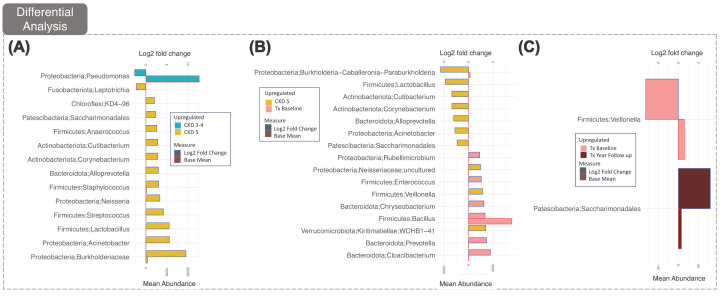
Differential Taxa analysis Genera found to be discriminately expressed based on differential taxa analyses showing which genera are up-/down-regulated between CKD 3-4 and CKD 5 (**A**), CKD 5 and KTx Baseline (**B**), and KTx baseline and KTx one year follow up (**C**), where they had at least a 2 log2 fold change from the mean abundance (Adj *P*-value ≤ 0.05). Left axis shows the range of Log2 fold change values whilst the right axis shows the mean abundance. In this manner, we can see whether the changes happen in low abundant or high abundant taxa.

Differential taxa analysis was then undertaken to investigate respective changes between all groups analysed (Figure 4). This indicated that only genus *Pseudomonas*, despite its presence in the *core microbiota* of both CKD5 and CKD3-4, was significantly increased in CKD3-4 (Figure 4A). Additionally, despite low mean abundances, *Neisseria, Streptococcus* and *Staphylococcus* were observed amongst the genera increased in the CKD5 vs CKD3-4 group; all are known TMA producers [11,13]. Similarly, the TMA producer Bacillus was identified in KTx baseline along with Prevotella, (Supplementary Figure S2). We observed no distinction between the core microbiotas of the KTx baseline and follow up group, indicating that KTx had no impact on core microbiota composition (Figure 3). Surprisingly, differential taxa analysis (Figure 4C) revealed only two genera showed significant changes, with Viellonella increased in KTx baseline and Saccharimonidales increasing after one year. We employed an additional analysis, QCAT-C, to consider the paired nature (i.e., originating from the same subject) of the KTx baseline and KTx follow-up samples. This detected no obvious changes between these two groups (Supplementary Figure S2). It did, however, show that there was a significant increase in the Firmicutes phylum in KTx compared with CKD3-4 and CKD5. At the genus level, Corynebacterium and Cutibacterium were significantly increased CKD3-4 and CKD5 versus KTx. The Bacilacaea family was increased in CKD3-4 versus CKD5.

### Determination of microbial community assembly processes in CKD and KTx

To determine how microbial communities in each of the respective groups were affected by their environment, we employed a null modelling approach (Supplementary Figure S3). Environmental filtering (Supplementary Figure S3a) showed that all groups observed clustering of their microbiota based on strong environmental pressure, as opposed to natural competition between species (NTI values all >2).

We then sought to investigate how the respective microbial communities were assembled in the respective cohorts, with a view to determining to what extent observed changes in the dysbiotic state were influenced by environmental selection (i.e., deterministic processes) or stoichastic processes (e.g., dispersal limitation in the environment, ecological drift, mutation), or a mix of both. We performed Quantitative process estimates (QPE) analysis (Supplementary Figure S3b), which classifies assembly processes in terms of selection (variable or homogenous), dispersal (dispersal limitation, or homogenising dispersal) or ‘undominated’ mechanisms. Differing environmental selective pressures result in extensive microbial compositional differences, due to variable, as opposed to homogeneous, selection. Similarly, limited, as opposed to homogenising dispersal, results in more dissimilar communities.

Our data clearly indicate that as the dysbiotic state progresses, composition of the CKD3-4 group is almost entirely dominated by stochastic processes, with undominated and homogenising dispersal accounting for 98% of total community assembly mechanisms observed. Variable selection accounted for the remaining 2%. Conversely, variable selection dominated the assembly processes in the CKD5 group, accounting for 68% of the overall structure. The remaining 33% was influenced by stochastic processes, particularly dispersal limitation (25%). The two KTx groups demonstrated largely uniform results, with a roughly 50:50 split between stochastic (51–54%) and deterministic (46–49%) approaches. Notably, the KTx follow-up group was the only group to exhibit any homogenising selection processes.

These observations were similarly supported by use of the Jaccard (incidence-based) metric (Supplementary data-see) indicating that there is a gradual shift in processes from determinism (CKD3-4) to stochasticism (KTx follow-up). Overall, this indicates that the community structures were largely deterministic (i.e. shaped by the uraemic environment), but after intervention, they were beginning to change to more stochastic assembly processes (i.e. a more normative state of assembly and maintenance).

As an additional metric for identifying microbial community assembly processes the Jaccard (incidence-based) metric was used to calculate normalised stochasticity ratio (NST) and modified stochasticity ratio (MST) (Supplementary Figure S3c). Both NST and MST showed that ratios for all groups were less than or equal to 50%, and so indicative of deterministic assembly processes overall. In all instances, CKD3-4 had the lowest NST and MST values (between 0.9 and 0.1), indicating the that these groups were most influenced by environmental pressures. The NST ratios of CKD5, and both KTx groups were ∼50% stochastic, indicating that there are a mix of stochastic and deterministic mechanisms involved in shaping the community. The MST ratios increased from CKD3-4 through to KTx follow-up for both P-P and P-F metrics, indicating that there was a gradual shift in processes from determinism (CKD3-4) to stochasticism (KTx follow-up).

### Analysis versus clinical metadata

PERMANOVA analysis (adjusted for FDR) of the associated clinical metadata is shown in Table 2. Notably, eGFR, diabetes, choline, betaine and TMAO all appeared to significantly impact the microbiome both in terms of community composition and function. The association of TMAO levels with both taxonomy (Weighted Unifrac 9%; *P*=0.001), and community function (12%; *P*=0.001) are noteworthy. The conversion of TMA to TMAO is reversible and can be catalysed by bacteria holding the capacity to code for TMAO reductase. *Pseudomonas, Bacillus* and *Staphylococcus* were all identified as prevalent genera in this study, and all possess TMAO reductase [11]. This is consistent with a thesis whereby a dysbiotic gut produces more TMAO, which then promotes an environment favouring the proliferation of bacteria which can produce TMA from TMAO. Medication and supplementation appeared to have the highest impact on the respective microbial communities’ composition and function. Vitamin D, ESA and phosphate binders explained 22%, 29% and 34% of the variability in the functional capacity of the respective microbial communities.

**Table 2:** PERMANOVA analysis explaining percentage variability (R2) due to different sources of variation considered in this study affecting composition, phylogeny, and function

Covariate	Bray Curtis	Unweighted UniFrac	Weighted UniFrac	Functional Hierarchial Meta-Storm
**Demographics**				
Age	NS	R2 = 0.02 *	NS	NS
Gender	NS	NS	NS	NS
Diabetes	R2 = 0.04 *	NS	NS	NS
**Biochemical parameters**				
S-albumin	NS	NS	NS	NS
Betaine	R2 = 0.11 ***	R2 = 0.02 ***	R2 = 0.17 ***	R2 = 0.10 ***
Choline	R2 = 0.14 ***	R2 = 0.03 ***	R2 = 0.17 ***	R2 = 0.14 ***
TMAO	R2 = 0.09 ***	R2 = 0.02 ***	R2 = 0.13 ***	R2 = 0.11 ***
Calcium	NS	R2 = 0.01 *	R2 = 0.03 ***	NS
Phosphate	R2 = 0.03 *	NS	NS	R2 = 0.04 *
Cholesterol	NS	R2 = 0.02 *	NS	NS
hsCRP	NS	NS	NS	NS
IL-6	R2 = 0.03 ***	R2 = 0.02 *	R2 = 0.04 *	NS
eGFR	R2 = 0.12 ***	R2 = 0.02 *	R2 = 0.06 **	R2 = 0.17 ***
**Drug treatments**				
Diuretics	NS	NS	NS	NS
Vitamin D	R2 = 0.21 **	NS	NS	R2 = 0.22 *
ESA	R2 = 0.26 **	R2 = 0.06 *	NS	R2 = 0.29 **
Phosphate binder	R2 = 0.32 **	R2 = 0.06 *	R2 = 0.08 *	R2 = 0.34 **
Statins	NS	NS	NS	NS

TMAO; trimethylamine N-oxide, IL; interleukin, ESA; erythropoietin stimulating agent *P<0.05, **P<0.01 and ***P<0.001.

### Analysis of one carbon metabolism

**Figure 5:**
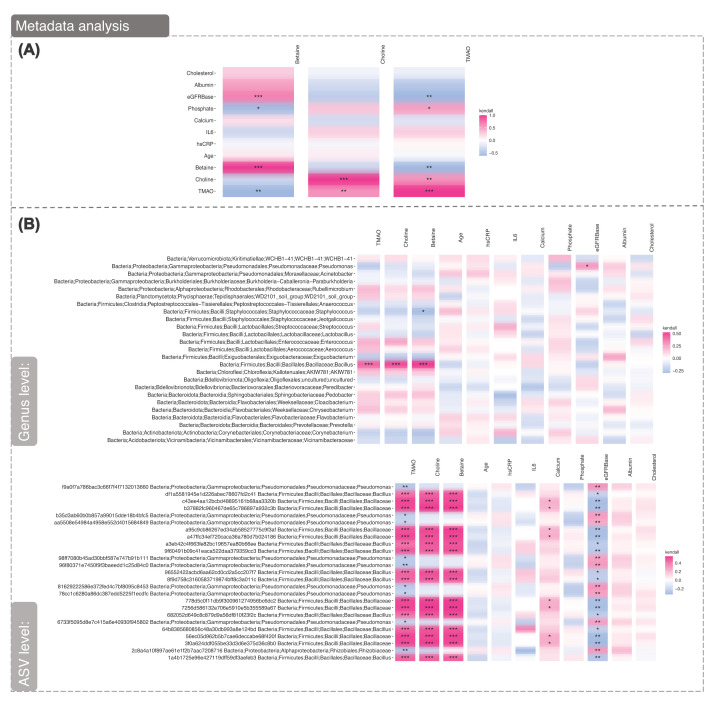
Correlation Analysis of Continuous variables within the Metadata (**A**) Kendall rank correlation analysis of metadata against elements of one-carbon metabolism, TMAO, Choline, Betaine. (**B**) Kendall Rank correlation analysis of the metadata against the 25 most abundant genera and ASVs. Note that metadata was only available for the baseline group of the KTx cohort, and so encompasses the CKD 5, CKD 3-4, and KTx baseline groups only. Bonferroni correction was used to adjust for multiple comparisons. *P* values <0.001 is denoted with (***), <0.01 with (**), <0.05 with (*) <0.1 with (.)

We have reported that CKD is a disease of accelerated ageing and that diminished renal function is associated with alterations in one carbon metabolism, especially lower betaine levels and an altered microbiome in the general population. We, therefore, used Kendall Rank correlation analysis (Figure 5) to determine if variables in the metadata correlated with elements of one-carbon metabolism (betaine, choline and TMAO) across the CKD and KTx groups. As expected, eGFR showed a significant positive correlation with betaine, whereas phosphate and TMAO showed inverse correlations. Choline showed significant positive correlation with TMAO only. TMAO positively associated with phosphate and choline, and negatively with betaine and eGFR. The same correlation analysis method was performed to determine associations between the metadata and the 25 most abundant genera across all samples (Figure 5B). This was also performed for ASVs, to elucidate any notable correlations not detected at the genus level. We found levels of *Pseudomonas* positively correlated with eGFR (*P*<0.05), and negatively with TMAO levels (*P*<0.05 to *P*<0.01), indicating a potential mechanism for *Pseudomonas*-mediated renal clearance of microbial metabolites. Conversely, increased *Bacilus/Bacilaceae* (dominant in KTx baseline), displayed a negative correlation with eGFR (*P*<0.05–0.01), but a positive correlation with TMAO, choline and betaine levels (*P*<0.001).

At KTx baseline, while Streptococcus and Chryseobacteria were positively associated with TMAO levels, while Bacillus and Pseudomonas displayed a negative association (Supplementary Figure S4). As Streptococcus displayed a negative correlation with eGFR, reduced renal clearance could contribute to the over-proliferation of Streptococcus, and an increase TMAO. In keeping with this Streptococcus and Cutibacterium both displayed a positive correlation with chronological age (Figure 4B) and inflammatory burden respectively (Supplementary Figure S4c).

### KEGG orthologic pathway analysis

The variability in genes contributing to specified KEGG Orthologic (KO) pathways is presented in Supplementary Figure S5. CKD3-4 displayed significantly increased variability in genes relating to pathways involved in anti-microbial resistance (AMR) and betaine biosynthesis. The two KTx groups displayed more variation in KOs relating to longevity pathways than the CKD groups. These data are again in keeping with the presence of more pro-inflammatory microbes in the CKD groups, as opposed to KTx where better physiological function is observed. Again, KO analyses emphasise the importance of one carbon metabolism and the capacity to synthesise betaine within the microbial community, given the relevance of betaine to maintenance of the epigenome via its part in the methionine cycle. Notably, KO analysis indicates that such a capacity is associated with better renal function and a more salutogenic community function following KTx.

## Discussion

Our data have exemplified ‘the Anna Karenina principle’ [14] whereby ‘*all happy families look alike; each unhappy family is unhappy in its own way’*, indicating that dysbiotic microbiotas of diseased individuals are more varied in their composition compared to those of healthy individuals. Our study has shown that the microbial taxonomic and functional diversity is significantly greater between individuals at CKD5, than at CKD3-4 or following KTx groups, which were more uniform. Notably, the respective *core microbiota* remained essentially the same after KTx (Figures 3 and 4B).

The results indicate that the progression of CKD from stages 3-4 to stage 5 coincides with increasing dysbiosis and the proliferation of bacteria capable of producing the pro-inflammatory metabolite TMA. Our findings from differential taxa analysis (Figure 4) showed that *Pseudomonas* was the only genus increased in abundance between CKD3-4 versus CKD5. Although being a putative TMA producer, its abundance was negatively correlated with TMAO levels (Supplementary Figure S3 and 4a). As reduced renal clearance is the main contributory factor to elevated TMAO levels in CKD [15], any increase in abundance of *Pseudomonas* and thus TMA, may be offset by increase in TMA utilisation by other community members. This remains to be proven.

Despite low mean abundances, other TMA producing pathobionts, such as *Neisseria, Streptococcus* and *Staphylococcus*, were among the genera increased at CKD5 (Figure 4a) [11,13,16–18]. *Pseudomonas* and *Bacillus* were particularly notable for their prevalence in all our analyses, and indeed our subset analysis showed that they have significant influence over the core microbiota, cementing themselves as putative key bacteria enabling CKD progression. Their dominance is pertinent to their TMA producing capability and associations with eGFR, suggesting that the microbiota is linked to the kidney’s ability to clear inflammatory metabolites. Another bacterium, Cutibacterium, showed a positive correlation with age (Supplementary Figure S4b) and IL-6 levels (Supplementary Figure S4c), consistent with a contribution to the inflammatory burden and the uremic premature ageing phenotype. We also provided evidence that the presence of known salutobionts correlated with better clinical characteristics in these cohorts, including the Nrf2 agonist producing Lactobacillus, which displayed a negative correlation with IL-6 (Supplementary Figure S4c). Similarly, Prevotella, which has salutogenic properties through its ability to produce SCFAs, was positively associated with eGFR (Supplemtary Figure S4b).

We observed, that despite an improvement in renal function following KTx, both core and discriminant microbiota analysis indicated little change between the genera present before and after KTx (Figures 3 and 4C). Although the microbiota might be expected to naturally restore itself through natural competition between species when renal function is partially restored, immunosuppressive treatment and intercurrent infectious events requiring antibiotic treatment may affect microbiotal composition after KTx [19–21]. Mounting evidence suggests that immunosuppressive treatment, especially corticosteroids [22], affect the gut microbiota [21]. Although it has been reported that immunosuppressants are associated with changes in gut flora, the impact on clinical outcomes is unknown [19]. Indeed, the benefits of a naturally evolving ecosystem, not driven by environmental pressures was not reflected in our core and differential microbiota analyses, which showed no clear benefit to the microbiota in terms of species present after KTx (Figure 2D). Taken together, while KTx improved renal function, the composition of the microbiome reflecting the burden of ‘wear and tear’ was not improved. Our observation highlights the importance of intervention studies to test whether dietary regimens (such as plant-based and fermented diets), probiotics, prebiotics and/or different immunosuppressive regimes can normalize the microbiota following a successful KTx.

Our data have highlighted the importance of the relationship between one carbon metabolism and the microbiome. Low betaine is a feature coincident with CKD, premature ageing, and a dysregulated microbiota [5,15]. Our metadata analysis confirmed that levels of TMAO, betaine and choline reflected variation in the microbiome and may be key to shaping the uremic microbiome (Table 2) and its links to nutrition and renal function. This link could be exploited in the development of targeted dietary intervention strategies [23], used alongside KRT, to alleviate the inflammatory load via the improvement of gut health. A number of studies have already successfully trialled betaine as a dietary intervention strategy to re-establish a more normative microbiome [24–26]. Additionally, the microbial *gbu* gene cluster has been identified as critical for the conversion dietary carnitine, via a γBB substrate, to TMA then TMAO in hosts, that contributes directly to CVD risk [27]. This again is targetable by a ‘food as medicine’ approach, which incorporates reduced carnitine intake by reduced red meat consumption.

We have shown that the microbiota of all groups investigated is subject to strong environmental pressure (Supplementary Figure S3), as opposed to natural competition between species. However, as differing selective pressures in different environments, results in extensive compositional differences in these microbial communities, our data provides novel insight into community *assembly* processes in CKD. Significantly, composition of the CKD3-4 group was dominated by stochastic processes and not environmental determinism. Stochasticity accounted for 98% of total community assembly in this group. Conversely, the CKD5 group was more deterministic in nature and dominated by variable selection (∼ 68% of the overall structure). The remainder (∼33%) was influenced by stochastic processes, notably dispersal limitation (25%). The KTx group was more uniform and displayed ∼ 50:50 split between stochastic (51–54%) and deterministic (46–49%) processes. Notably, the KTx follow-up group was the only group to exhibit any homogenising selection processes. These findings are supported by our additional analyses and reinforce the idea that CKD5 patients have a significantly dysfunctional microbiota shaped by an elevated uraemic milieu and associated with an imminent threshold of critical loss of renal function. Following KTx the microbiota appears to show more similarities with incident dialysis patients, with respect to how the microbial is assembled. It is also more salutogenic.

Linked to our community assembly analyses, functional analysis of KO pathways was used to investigate how community composition affected possible functional capabilities (Supplementary Figure S4). This revealed that genes relating to longevity and one carbon metabolism in the KTx group caused more variation to the microbiota diversity compared to CKD3-4 and CKD5 patients. This could indicate that these pathways promote a healthier landscape of ageing following KTx and amelioration of the effects of the dysbiotic microbome at CKD5.

Our study has a range of advantages and limitations which need consideration. We would contend that our analyses of the circulatory signature of bacterial DNA fragments escaping through a leaky gut, or via the oral mucosal surfaces, gives potentially a more comprehensive picture of the bacteria influencing human health than the oral cavity or gut alone. The rather large number and prospective analyses of patients undergoing LD-KTx also benefits the study. However, several limitations should also be considered. Firstly, these include the relatively small numbers of CKD3-4 and CKD5 patients, and a sex imbalance in favour of males (as usually seen in CKD). Secondly, patients selected for LD-KTx were a selected group of younger and healthier CKD5 patients and do not represent the typical CKD5 patient. Thirdly, a formal demonstration of which cavity (e.g. gut, oral) bacterial DNA signatures in the circulation originated from would be informative, especially in relation to the degree of gut leakiness. Finally, as diet affect both gut microbial composition and the generation of TMA and TMAO in the uremic milieu [28] information on long-term nutritional habits would have benefited the study. This has direct implications for future patient management.

## Clinical perspectives

- It remains to be determined if kidney replacement therapy mitigate microbial dysbiosis and the production of inflammatory microbial metabolites in the uraemic milieu.
- A normative microbiome is not restored following kidney transplantation. Metadata analysis confirmed that TMAO had significant impact upon both inflammatory burden and the composition of the microbiome.
- TMAO is key to shaping the uraemic microbiome and might be exploited in the development of dietary intervention strategies to enable the restoration of a more salutogenic microbiome.

## Supplementary Material

Supplementary Figures S1-S5 and Supplementary Data Materials

## Data Availability

All relevant patient data are stored anonymised and are available upon request in compliance with ethical permission. Data from this study are available from the senior authors.

## Open Access

Open access for this article was enabled by the participation of Karolinska Institute in an all-inclusive *Read & Publish* agreement with Portland Press and the Biochemical Society under a transformative agreement with Individual.

## Abbreviations

AMR: anti-microbial resistance
CKD: chronic kidney disease
KRT: kidney replacement therapies
KTx: kidney transplantation
Nrf2: nuclear factor erythroid 2-related factor 2
TMA: trimethylamine
TMAO: trimethylamine N-oxide
