# Supplementary material for: A normative microbiome is not restored following kidney transplantation
Source: Clin Sci (Lond). 2023 Oct 17;137(20):1563–75. doi: 10.1042/CS20230779 (PMC10582644; doi:10.1042/CS20230779)
Supplement: Supplementary Figures S1-S5 and Supplementary Data Materials [file CS-2023-0779_supp.pdf]

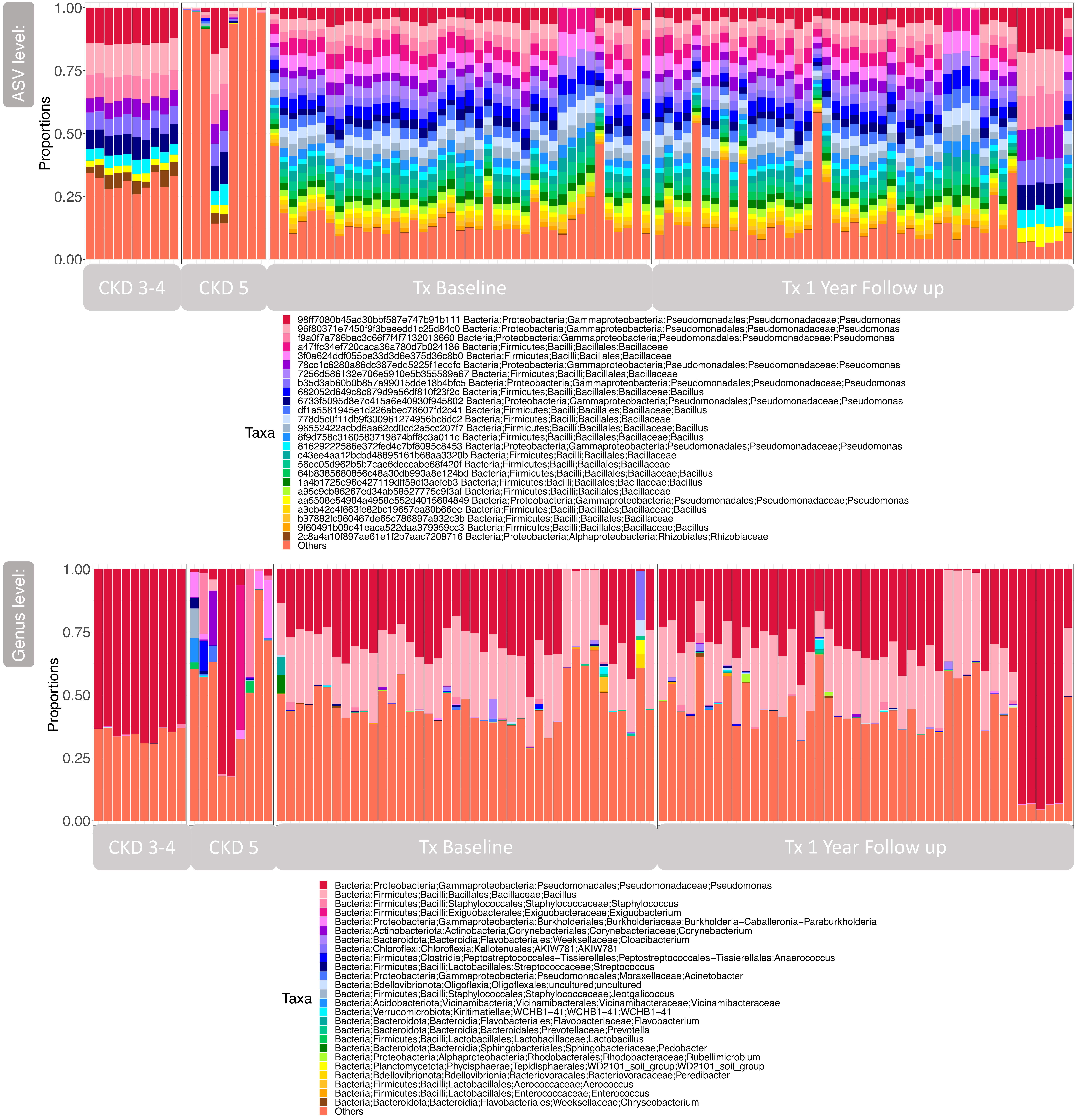
ASV level:
Proportions
1.00
0.75
0.50
0.25
0.00
CKD 3-4
CKD 5
Tx Baseline
Tx 1 Year Follow up
Taxa
98ff7080b45ad30bbf587e747b91b111 Bacteria;Proteobacteria;Gammaproteobacteria;Pseudomonadales;Pseudomonadaceae;Pseudomonas
96f80371e7450f9f3baeedd1c25d84c0 Bacteria;Proteobacteria;Gammaproteobacteria;Pseudomonadales;Pseudomonadaceae;Pseudomonas
f9a0f7a786bac3c66f7f4f7132013660 Bacteria;Proteobacteria;Gammaproteobacteria;Pseudomonadales;Pseudomonadaceae;Pseudomonas
a47ffc34ef720caca36a780d7b024186 Bacteria;Firmicutes;Bacilli;Bacillales;Bacillaceae
3f0a624ddf055be33d3d6e375d36c8b0 Bacteria;Firmicutes;Bacilli;Bacillales;Bacillaceae
78cc1c6280a86dc387edd5225f1ecdfc Bacteria;Proteobacteria;Gammaproteobacteria;Pseudomonadales;Pseudomonadaceae;Pseudomonas
7256d586132e706e5910e5b355589a67 Bacteria;Firmicutes;Bacilli;Bacillales;Bacillaceae
b35d3ab60b0b857a99015dde18b4bfc5 Bacteria;Proteobacteria;Gammaproteobacteria;Pseudomonadales;Pseudomonadaceae;Pseudomonas
682052d649c8c879d9a56df810f23f2c Bacteria;Firmicutes;Bacilli;Bacillales;Bacillaceae;Bacillus
6733f5095d8e7c415a6e40930f945802 Bacteria;Proteobacteria;Gammaproteobacteria;Pseudomonadales;Pseudomonadaceae;Pseudomonas
df1a5581945e1d226abec78607fd2c41 Bacteria;Firmicutes;Bacilli;Bacillales;Bacillaceae;Bacillus
778d5c0f11db9f300961274956bc6dc2 Bacteria;Firmicutes;Bacilli;Bacillales;Bacillaceae
96552422acbd6aa62cd0cd2a5cc207f7 Bacteria;Firmicutes;Bacilli;Bacillales;Bacillaceae;Bacillus
8f9d758c3160583719874bff8c3a011c Bacteria;Firmicutes;Bacilli;Bacillales;Bacillaceae;Bacillus
81629222586e372fed4c7bf8095c8453 Bacteria;Proteobacteria;Gammaproteobacteria;Pseudomonadales;Pseudomonadaceae;Pseudomonas
c43ee4aa12bcbd48895161b68aa3320b Bacteria;Firmicutes;Bacilli;Bacillales;Bacillaceae
56ec05d962b5b7cae6deccabe68f420f Bacteria;Firmicutes;Bacilli;Bacillales;Bacillaceae
64b8385680856c48a30db993a8e124bd Bacteria;Firmicutes;Bacilli;Bacillales;Bacillaceae;Bacillus
1a4b1725e96e427119dff59df3aefeb3 Bacteria;Firmicutes;Bacilli;Bacillales;Bacillaceae;Bacillus
a95c9cb86267ed34ab58527775c9f3af Bacteria;Firmicutes;Bacilli;Bacillales;Bacillaceae
aa5508e54984a4958e552d4015684849 Bacteria;Proteobacteria;Gammaproteobacteria;Pseudomonadales;Pseudomonadaceae;Pseudomonas
a3eb42c4f663fe82bc19657ea80b66ee Bacteria;Firmicutes;Bacilli;Bacillales;Bacillaceae;Bacillus
b37882fc960467de65c786897a932c3b Bacteria;Firmicutes;Bacilli;Bacillales;Bacillaceae
9f60491b09c41eaca522daa379359cc3 Bacteria;Firmicutes;Bacilli;Bacillales;Bacillaceae;Bacillus
2c8a4a10f897ae61e1f2b7aac7208716 Bacteria;Proteobacteria;Alphaproteobacteria;Rhizobiales;Rhizobiaceae
Others
Genus level:
Proportions
1.00
0.75
0.50
0.25
0.00
CKD 3-4
CKD 5
Tx Baseline
Tx 1 Year Follow up
Taxa
Bacteria;Proteobacteria;Gammaproteobacteria;Pseudomonadales;Pseudomonadaceae;Pseudomonas
Bacteria;Firmicutes;Bacilli;Bacillales;Bacillaceae;Bacillus
Bacteria;Firmicutes;Bacilli;Staphylococcales;Staphylococcaceae;Staphylococcus
Bacteria;Firmicutes;Bacilli;Exiguobacterales;Exiguobacteraceae;Exiguobacterium
Bacteria;Proteobacteria;Gammaproteobacteria;Burkholderiales;Burkholderiaceae;Burkholderia−Caballeronia−Paraburkholderia
Bacteria;Actinobacteriota;Actinobacteria;Corynebacteriales;Corynebacteriaceae;Corynebacterium
Bacteria;Bacteroidota;Bacteroidia;Flavobacteriales;Weeksellaceae;Cloacibacterium
Bacteria;Chloroflexi;Chloroflexia;Kallotenuales;AKIW781;AKIW781
Bacteria;Firmicutes;Clostridia;Peptostreptococcales−Tissierellales;Peptostreptococcales−Tissierellales;Anaerococcus
Bacteria;Firmicutes;Bacilli;Lactobacillales;Streptococcaceae;Streptococcus
Bacteria;Proteobacteria;Gammaproteobacteria;Pseudomonadales;Moraxellaceae;Acinetobacter
Bacteria;Bdellovibrionota;Oligoflexia;Oligoflexales;uncultured;uncultured
Bacteria;Firmicutes;Bacilli;Staphylococcales;Staphylococcaceae;Jeotgalicoccus
Bacteria;Acidobacteriota;Vicinamibacteria;Vicinamibacterales;Vicinamibacteraceae;Vicinamibacteraceae
Bacteria;Verrucomicrobiota;Kiritimatiellae;WCHB1−41;WCHB1−41;WCHB1−41
Bacteria;Bacteroidota;Bacteroidia;Flavobacteriales;Flavobacteriaceae;Flavobacterium
Bacteria;Bacteroidota;Bacteroidia;Bacteroidales;Prevotellaceae;Prevotella
Bacteria;Firmicutes;Bacilli;Lactobacillales;Lactobacillaceae;Lactobacillus
Bacteria;Bacteroidota;Bacteroidia;Sphingobacteriales;Sphingobacteriaceae;Pedobacter
Bacteria;Proteobacteria;Alphaproteobacteria;Rhodobacterales;Rhodobacteraceae;Rubellimicrobium
Bacteria;Planctomycetota;Phycisphaerae;Tepidisphaerales;WD2101_soil_group;WD2101_soil_group
Bacteria;Bdellovibrionota;Bdellovibrionia;Bacteriovoracales;Bacteriovoracaceae;Peredibacter
Bacteria;Firmicutes;Bacilli;Lactobacillales;Aerococcaceae;Aerococcus
Bacteria;Firmicutes;Bacilli;Lactobacillales;Enterococcaceae;Enterococcus
Bacteria;Bacteroidota;Bacteroidia;Flavobacteriales;Weeksellaceae;Chryseobacterium
Others

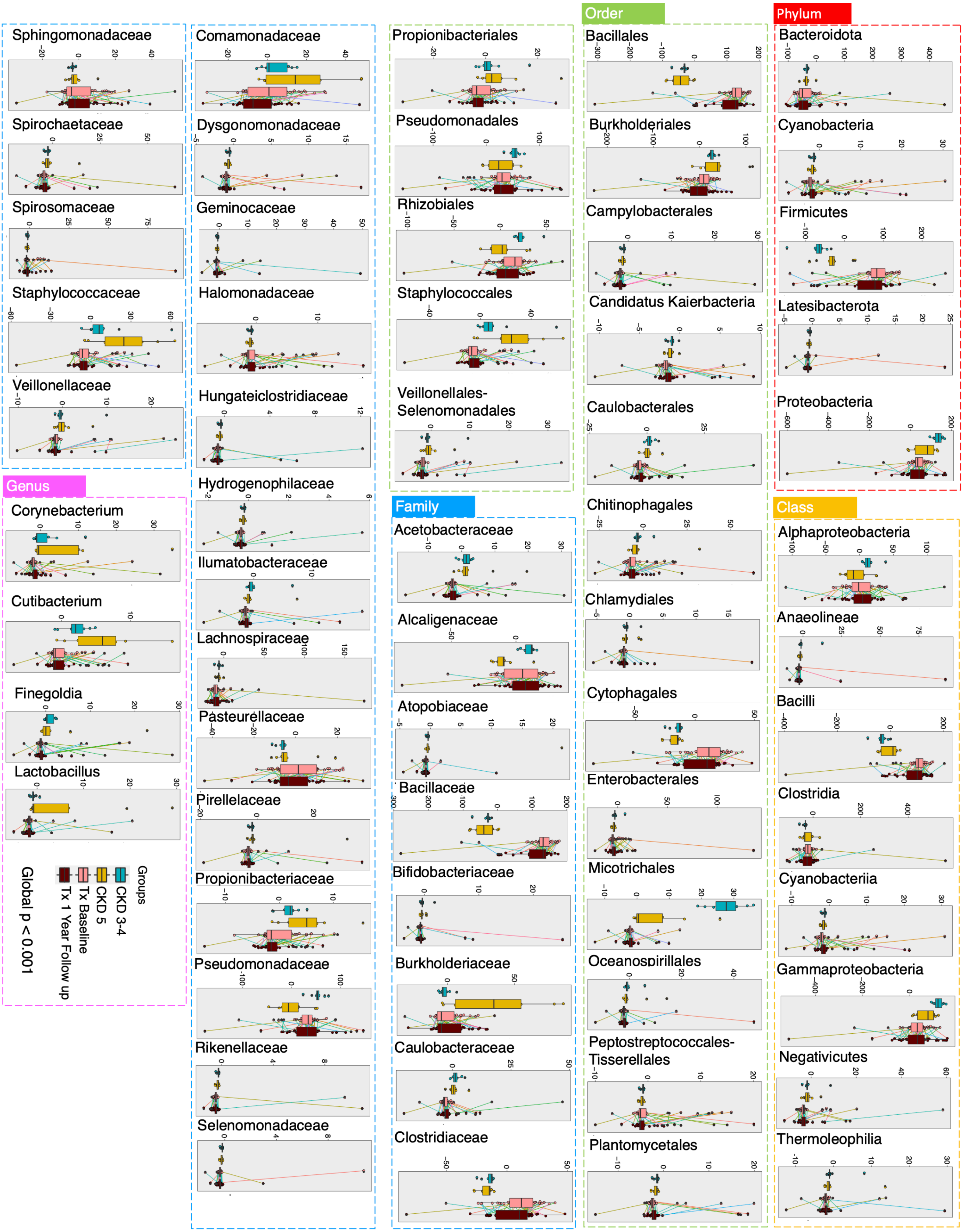
Phylum
Bacteroidota
Cyanobacteria
Firmicutes
Latesibacterota
Proteobacteria
Class
Alphaproteobacteria
Anaerolineae
Bacilli
Clostridia
Cyanobacteriia
Gammaproteobacteria
Negativicutes
Thermoleophilia
Order
Bacillales
Burkholderiales
Campylobacterales
Candidatus Kaierbacteria
Caulobacterales
Chitinophagales
Chlamydiales
Cytophagales
Enterobacterales
Micotrichales
Oceanospirillales
Peptostreptococcales-Tissierellales
Plantomycetales
Propionibacteriales
Pseudomonadales
Rhizobiales
Staphylococcales
Veillonellales-Selenomonadales
Family
Acetobacteraceae
Alcaligenaceae
Atopobiaceae
Bacillaceae
Bifidobacteriaceae
Burkholderiaceae
Caulobacteraceae
Clostridiaceae
Comamonadaceae
Dysgonomonadaceae
Geminocaceae
Halomonadaceae
Hungateiclostridiaceae
Hydrogenophilaceae
Ilumatobacteraceae
Lachnospiraceae
Pasteurellaceae
Pirellelaceae
Propionibacteriaceae
Pseudomonadaceae
Rikenellaceae
Selenomonadaceae
Sphingomonadaceae
Spirochaetaceae
Spirosomaceae
Staphylococcaceae
Veillonellaceae
Genus
Corynebacterium
Cutibacterium
Finegoldia
Lactobacillus
Groups
CKD 3-4
CKD 5
Tx Baseline
Tx 1 Year Follow up
Global p < 0.001

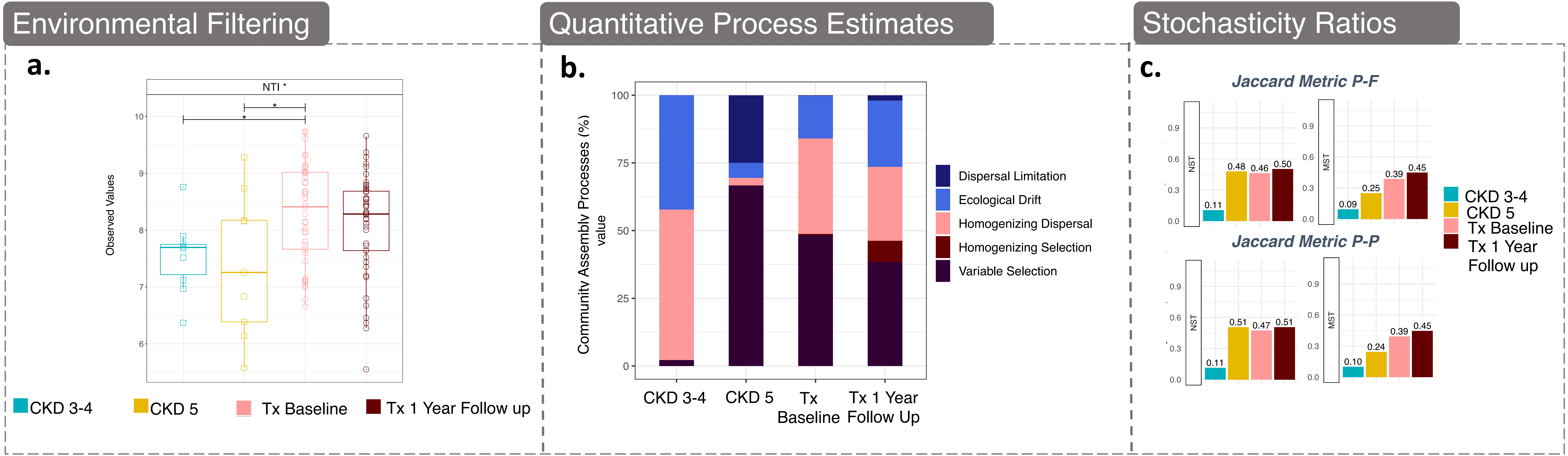

Environmental Filtering
Quantitative Process Estimates
Stochasticity Ratios
a.
NTI *
Observed Values
CKD 3-4
CKD 5
Tx Baseline
Tx 1 Year Follow up
b.
Community Assembly Processes (%)
value
Dispersal Limitation
Ecological Drift
Homogenizing Dispersal
Homogenizing Selection
Variable Selection
CKD 3-4
CKD 5
Tx Baseline
Tx 1 Year Follow Up
c.
Jaccard Metric P-F
NST
MST
0.11
0.48
0.46
0.50
0.09
0.25
0.39
0.45
Jaccard Metric P-P
0.11
0.51
0.47
0.51
0.10
0.24
0.39
0.45
CKD 3-4
CKD 5
Tx Baseline
Tx 1 Year Follow up

Tx Baseline

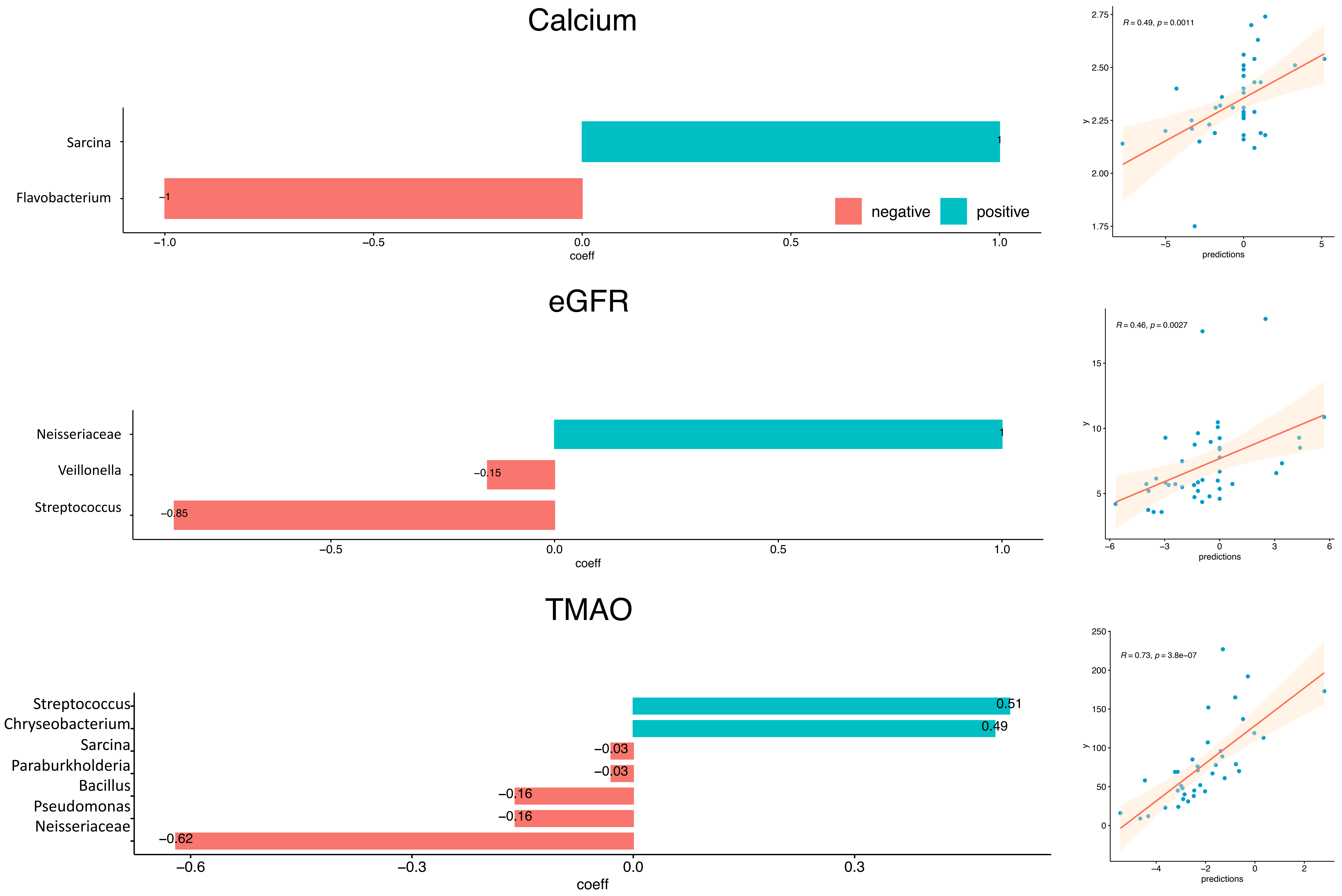

Calcium
Sarcina
Flavobacterium
1
−1
negative
positive
−1.0
−0.5
0.0
0.5
1.0
coeff
R = 0.49, p = 0.0011
2.75
2.50
2.25
2.00
1.75
y
−5
0
5
predictions
eGFR
Neisseriaceae
Veillonella
Streptococcus
1
−0.15
−0.85
−0.5
0.0
0.5
1.0
coeff
R = 0.46, p = 0.0027
15
10
5
y
−6
−3
0
3
6
predictions
TMAO
Streptococcus
Chryseobacterium
Sarcina
Paraburkholderia
Bacillus
Pseudomonas
Neisseriaceae
0.51
0.49
−0.03
−0.03
−0.16
−0.16
−0.62
−0.6
−0.3
0.0
0.3
coeff
R = 0.73, p = 3.8e−07
250
200
150
100
50
0
y
−4
−2
0
2
predictions

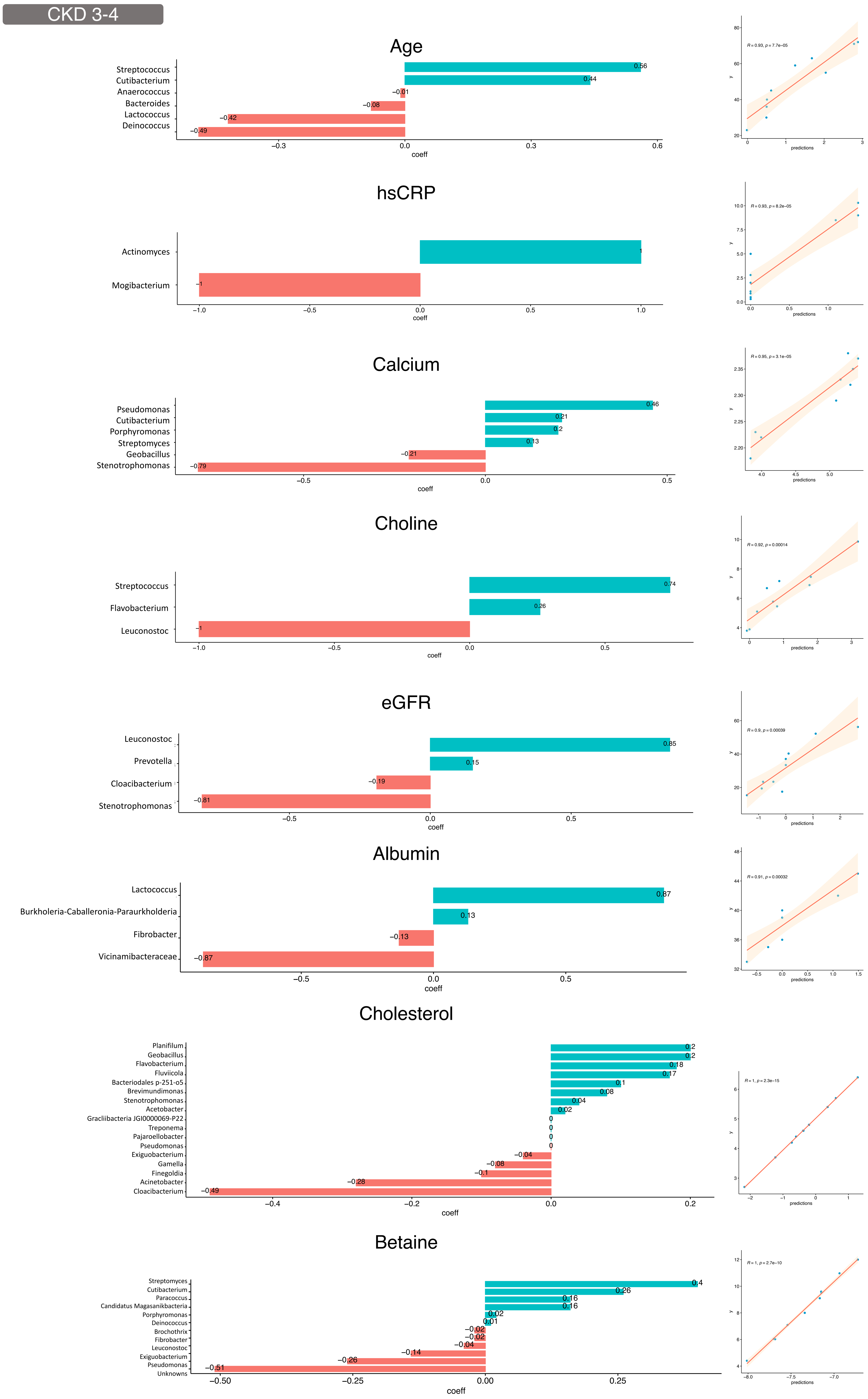

CKD 3-4
Age
Streptococcus 0.56
Cutibacterium 0.44
Anaerococcus −0.01
Bacteroides −0.08
Lactococcus −0.42
Deinococcus −0.49
coeff
R = 0.93, p = 7.7e−05
predictions
hsCRP
Actinomyces 1
Mogibacterium −1
coeff
R = 0.93, p = 8.2e−05
predictions
Calcium
Pseudomonas 0.46
Cutibacterium 0.21
Porphyromonas 0.2
Streptomyces 0.13
Geobacillus −0.21
Stenotrophomonas −0.79
coeff
R = 0.95, p = 3.1e−05
predictions
Choline
Streptococcus 0.74
Flavobacterium 0.26
Leuconostoc −1
coeff
R = 0.92, p = 0.00014
predictions
eGFR
Leuconostoc 0.85
Prevotella 0.15
Cloacibacterium −0.19
Stenotrophomonas −0.81
coeff
R = 0.9, p = 0.00039
predictions
Albumin
Lactococcus 0.87
Burkholeria-Caballeronia-Paraurkholderia 0.13
Fibrobacter −0.13
Vicinamibacteraceae −0.87
coeff
R = 0.91, p = 0.00032
predictions
Cholesterol
Planifilum 0.2
Geobacillus 0.2
Flavobacterium 0.18
Fluviicola 0.17
Bacteriodales p-251-o5 0.1
Brevimundimonas 0.08
Stenotrophomonas 0.04
Acetobacter 0.02
Gracilibacteria JGI0000069-P22 0
Treponema 0
Pajaroellobacter 0
Pseudomonas 0
Exiguobacterium −0.04
Gamella −0.08
Finegoldia −0.1
Acinetobacter −0.28
Cloacibacterium −0.49
coeff
R = 1, p = 2.3e−15
predictions
Betaine
Streptomyces 0.4
Cutibacterium 0.26
Paracoccus 0.16
Candidatus Magasanikbacteria 0.16
Porphyromonas 0.02
Deinococcus 0.01
Brochothrix −0.02
Fibrobacter −0.02
Leuconostoc −0.04
Exiguobacterium −0.14
Pseudomonas −0.26
Unknowns −0.51
coeff
R = 1, p = 2.7e−10
predictions

CKD 5

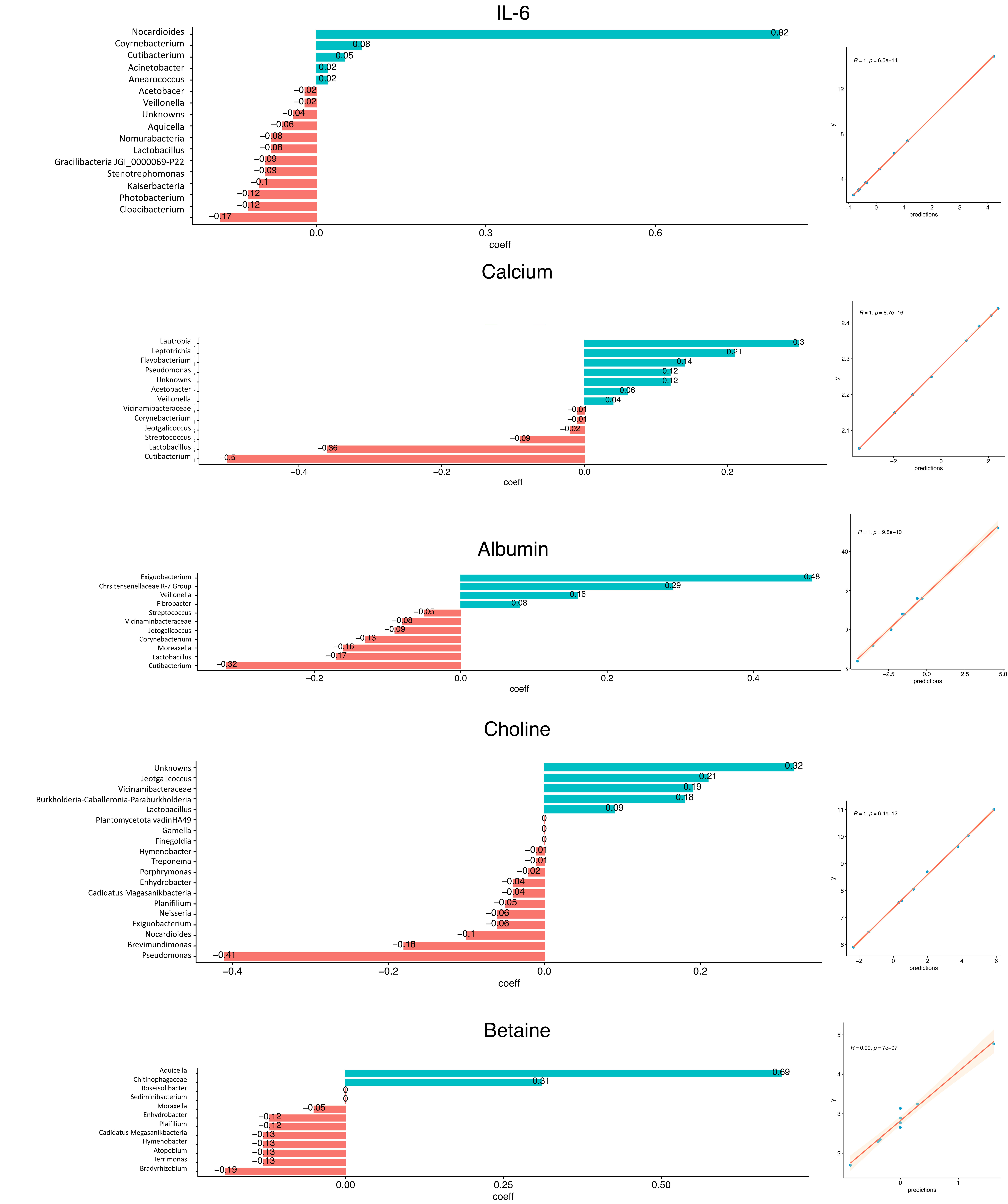

IL-6
Nocardioides 0.82
Coyrnebacterium 0.08
Cutibacterium 0.05
Acinetobacter 0.02
Anearococcus 0.02
Acetobacer −0.02
Veillonella −0.02
Unknowns −0.04
Aquicella −0.06
Nomurabacteria −0.08
Lactobacillus −0.08
Gracilibacteria JGI_0000069-P22 −0.09
Stenotrephomonas −0.09
Kaiserbacteria −0.1
Photobacterium −0.12
Cloacibacterium −0.12
−0.17
0.0
0.3
0.6
coeff
R = 1, p = 6.6e−14
y
predictions
Calcium
Lautropia 0.3
Leptotrichia 0.21
Flavobacterium 0.14
Pseudomonas 0.12
Unknowns 0.12
Acetobacter 0.06
Veillonella 0.04
Vicinamibacteraceae −0.01
Corynebacterium −0.01
Jeotgalicoccus −0.02
Streptococcus −0.09
Lactobacillus −0.36
Cutibacterium −0.5
−0.4
−0.2
0.0
0.2
coeff
R = 1, p = 8.7e−16
y
predictions
Albumin
Exiguobacterium 0.48
Chrsitensenellaceae R-7 Group 0.29
Veillonella 0.16
Fibrobacter 0.08
Streptococcus −0.05
Vicinaminbacteraceae −0.08
Jetogalicoccus −0.09
Corynebacterium −0.13
Moreaxella −0.16
Lactobacillus −0.17
Cutibacterium −0.32
−0.2
0.0
0.2
0.4
coeff
R = 1, p = 9.8e−10
predictions
Choline
Unknowns 0.32
Jeotgalicoccus 0.21
Vicinamibacteraceae 0.19
Burkholderia-Caballeronia-Paraburkholderia 0.18
Lactobacillus 0.09
Plantomycetota vadinHA49 0
Gamella 0
Finegoldia 0
Hymenobacter −0.01
Treponema −0.01
Porphrymonas −0.02
Enhydrobacter −0.04
Cadidatus Magasanikbacteria −0.04
Planifilium −0.05
Neisseria −0.06
Exiguobacterium −0.06
Nocardioides −0.1
Brevimundimonas −0.18
Pseudomonas −0.41
−0.4
−0.2
0.0
0.2
coeff
R = 1, p = 6.4e−12
y
predictions
Betaine
Aquicella 0.69
Chitinophagaceae 0.31
Roseisolibacter 0
Sediminibacterium 0
Moraxella −0.05
Enhydrobacter −0.12
Plaifilium −0.12
Cadidatus Megasanikbacteria −0.13
Hymenobacter −0.13
Atopobium −0.13
Terrimonas −0.13
Bradyrhizobium −0.19
0.00
0.25
0.50
coeff
R = 0.99, p = 7e−07
y
predictions

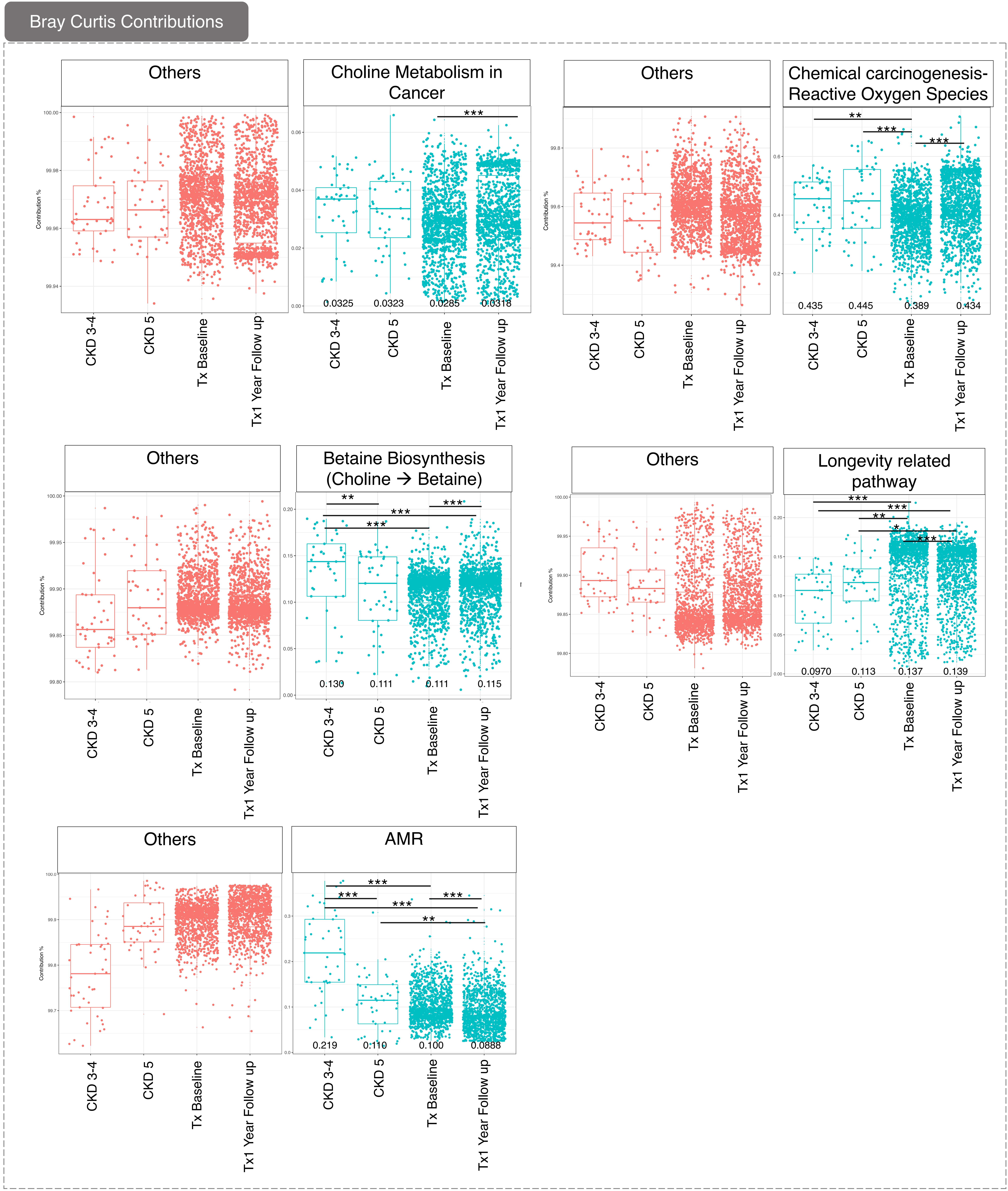

Bray Curtis Contributions
Others
Choline Metabolism in Cancer
Others
Chemical carcinogenesis-Reactive Oxygen Species
Contribution %
0.0325
0.0323
0.0285
0.0318
0.435
0.445
0.389
0.434
Others
Betaine Biosynthesis (Choline → Betaine)
Others
Longevity related pathway
0.130
0.111
0.111
0.115
0.0970
0.113
0.137
0.139
Others
AMR
0.219
0.110
0.100
0.0888
CKD 3-4
CKD 5
Tx Baseline
Tx1 Year Follow up

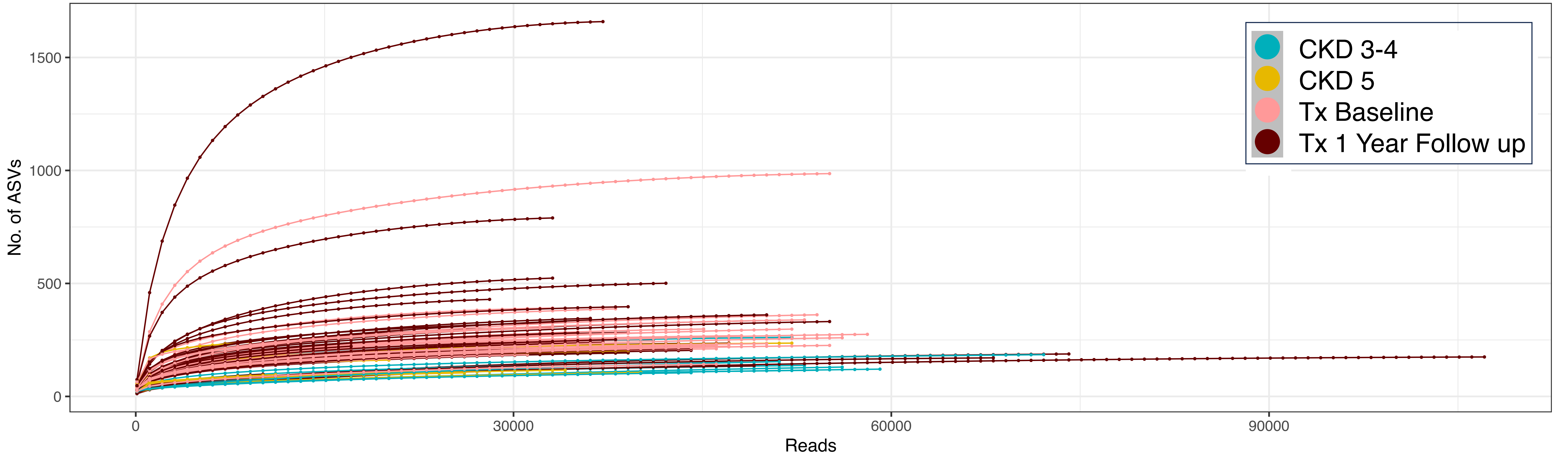
CKD 3-4
CKD 5
Tx Baseline
Tx 1 Year Follow up
No. of ASVs
1500
1000
500
0
0
30000
60000
90000
Reads

Methods

DNA isolation and 16S amplicon library preparation for microbiome analysis

DNA was extracted from peripheral blood leukocytes using Maxwell®16 System (Maxwell® 16 Blood DNA Purification kit, Promega), and quantified using the High Sensitivity DNA Qubit system (ThermoFisher, Paisley, UK). 16S libraries encompassing the V3-V4 regions were generated by Glasgow Polyomics as done in(Craven et al. 2021). Briefly, the V3 to V4 regions of bacterial 16S were amplified using Kapa HiFi Hotstart Readymix (2×) (Kapa Biosystems, Wilmington, MA, USA) with the addition of primers specific for the V3 and V4 regions of 16S (based on the standard Illumina 16S primers), which contain an overlap sequence making the primers compatible with the Nextera XT indexing reagents (Illumina, San Diego, CA, USA). Samples were then amplified using a 5 min 95 °C hotstart followed by 26 cycles of 95 °C for 30 s and 60 °C for 1 min with a final elongation step of 60 °C for 5 min. The resulting amplicons were purified using bead extraction (SPRI select beads, Beckman Coulter, Brea, CA, USA), using 0.9× beads followed by 80% ethanol washes and resuspension in 10 mM Tris-EDTA buffer. The amplicons were quantified using the High Sensitivity DNA Qubit system and profiles were obtained from an Agilent 2100 Bioanalyser using High Sensitivity DNA reagents (Agilent, Santa Clara, CA, USA). Samples were then standardized to 10 ng per reaction and amplified in the presence of Nextera XT v2 indexes using Kapa HiFi Hotstart readymix (2×) for 8 cycles. The resulting indexed libraries were then purified and quality controlled as before. The libraries were combined in equimolar ratios and sequenced on a MiSeq (Illumina, San Diego, CA, USA) instrument using a paired end, 2 × 300 bp, sequencing run. Samples were sequenced with an average of 50 000 reads per sample. Possible contamination of reagents was controlled for by running a negative control sample (Nuclease-Free water (Ambion™, AM9932, Thermo Fisher Scientific)), instead of a DNA sample through the whole analysis, in conjunction with the true test samples. Water only samples were treated identically to test samples.

Bioinformatics

Sequence Quality trimming and ASV Generation

Amplicon sequence variants (ASVs) were constructed, and abundance tables generated using the Qiime2 workflow. A total of 1348 ASVs were identified. Briefly, Paired-end reads were trimmed where the sequence quality dropped using the Deblur algorithm. Qiime phylogeny was used to generate the rooted phylogenetic tree for the ASVs, which were assigned taxonomy by classifying against the SILVA132 reference database. The abundance tables of ASVs were then combined with their taxonomy to generate the Biom file used for downstream statistical analysis in R.

Statistical Analyses

Statistical analyses were performed in R as by Ijaz, U.Z. *et al*, *2018*(Ijaz et al. 2018). The frequency based method from the decontam package was used to remove contaminating DNA which may have been introduced to samples in the library preparation process.

Alpha Diversity and Beta Diversity

The vegan package was used for alpha and beta diversity analyses. For alpha diversity measures we have used: Shannon entropy – a commonly used index to measure balance within a community, rarefied richness (exponential of Shannon entropy) – the estimated number of species, and Pielou's Evenness- A measure of abundance of each species present in a community ranging from 0 (not even, communities are dominated by particular species) to 1(completely even, abundance counts are evenly distributed amongst all species in the community). Ordination of ASV table in reduced space (beta diversity) was done using Principal Coordinate Analysis (PCoA) plots of ASVs using three different distance measures: Bray-Curtis (shown), Unweighted Unifrac and Weighted Unifrac. Unifrac distances were calculated using the phyloseq package (McMurdie and Holmes 2013). Analysis of variance for explanatory variables (or sources of variation) was performed using Vegan's adonis() against distance matrices (Bray-Curtis/UnweightedUniFrac/Weighted UniFrac/hierarchical MetaStorm). This function, referred to as PERMANOVA, fits linear models to distance matrices and used a permutation test with pseudo-F ratios to explain variability in the microbial community structure, contingent upon the variation in the given extrinsic parameter of interest.

Hierarchical MetaStorm (Functional dissimilarity measure)

As opposed to taxonomic beta diversity calculation which, assumes each feature (microbe) to be an independent entity, and traditionally uses Bray-Curtis distance (or any other count measure), calculation of functional beta diversity is challenging because of the presence of dependencies as well as redundancies in features (KOs). Therefore, applying traditional measures such as Bray-Curtis distance is not recommended as it leads to erroneous results. To capture redundancies as well as hierarchical nature of KOs which at higher levels form pathways, we used Hierarchical Meta-Storms (HMS),(Y. Zhang et al., 2021) which collapses the metabolic pathways at the observed KOs level by considering BRITE pathways as a set of reference pathways, and then propagating the abundances upward for these pathways in a multi-level pathway hierarchy to give a weighted dissimilarity measure. This then provides higher sensitivity for detecting variations in upper-level metabolic pathways between samples.

Subset Analysis

The sinkr package was used to implement the BVSTEP algorithm to search for the highest correlation (Mantel test) between dissimilarities in a fixed and variable multivariable dataset. Essentially, the method calculates original distances by determining the Bray-Curtis distance between samples using all the ASVs. It then permutes through the subset of ASVs, calculating the Bray-Curtis distances between the samples again for each permutation. These are then correlated against the original recorded distances until subsets are obtained that explain roughly the same beta diversity as the full set of ASVs. The algorithm permutes through 2n-1 possible combinations of ASVs features in the variable dataset. The 2000 most abundant ASVs were used for the correlation to identify the ASVs that were causing the most significant shifts in beta diversity, as being the most abundant we can assume that they have a significant role to play and will best correlate with the overall dissimilarities given if the total number of ASVs were used.

Null Modelling

A null modelling approach was used to determine the ecological processes driving community assembly, as described fully in (Trego et al. 2021). Briefly, Nearest Taxon Index (NTI) was used

to determine to what extent the communities were influenced by environmental pressures. This was done using the picante package (Kembel et al. 2010). Values >+2 indicate strong environmental pressure influencing the community, whereas values <-2 suggest that the driver is natural competition amongst species. The Jaccard (incidence-based) metric was used to calculate Normalised stochasticity ratio (NST) and modified stochasticity ratio (MST). Proportional-proportional(P-P) and proportional-fixed (P-F) taxa-richness constraints were applied for each metric used (Ning et al. 2019). Quantitative process estimates (QPE) method was also used as part of this approach, providing a breakdown of the assembly processes used to establish community structre. These can be broken down by selection processes (variable or homogenous), dispersal processes (dispersal limitation, or homogenising dispersal), or 'ecological drift' (undominated mechanisms) (Stegen et al. 2015; Vellend 2010). Communities which are affected strongly by selection pressures are most influenced by their environment. In this context, variable selection leads to a more varied community as a result of a multitude of environmental conditions being present, whereas homogenous selection gives rise to a more consistent community due an unchanging environment. Conversely, dispersal processes refer to the migration of organisms form one space to another. For example, high rates of dispersal within microbial communities is known as homogenising dispersal, and results in similar communities, whereas minimal movement of organisms (dispersal limitation) results in dissimilar communities. This type of dispersal can lead to ecological drift, a stochastic assembly processes (Trego et al. 2021).

Core Microbiome Analysis

To identify core microbiome, we have used the approach discussed in (Shade, A., & Stopnisek, N. (2019). Abundance-occupancy distributions to prioritize plant core microbiome membership. Current opinion in microbiology, 49, 50-58). The approach first ranks the ASVs by occupancy, and then calculates the minimal occupancy threshold dynamically by learning from the data. After ranking the ASVs, the subset of core taxa is constructed incrementally by adding one ASV at a time to the core set of ASVs, from highly prevalent to lowly prevalent ones. The contribution of the core subset to beta diversity is then calculated every time a new ASV becomes member of the core set using the Bray-Curtis distance in the equation, $C = 1 - \frac{BC_{core}}{BC_{all}}$. As per original author's recommendation, we have used an approach where the

occupancy threshold is decided by stopping when addition of an ASV does not cause more than 2% increase in the explanatory power by Bray-Curtis distance. Independently, a neutral model (Shade, A., & Stopnisek, N. (2019). Abundance-occupancy distributions to prioritize plant core microbiome membership. Current opinion in microbiology, 49, 50-58). is fitted to the "S" shaped abundance-occupancy distributions informing about the OTUs that are likely selected by the environment. These are obtained as those that fall outside the 95% confidence interval of the fitted model, and are inferred to be deterministically assembled, rather than neutrally selected, with those that are *above the model selected by the host environment* (represented by red colour), and those points below the model *are dispersal limited* (represented by blue colour). We have used site-specific occupancy, where occupancy is viewed as a detection within a particular cohort (CKD 3-4, CKD 5, TX Baseline, TX Year Followup), such that as long as the ASVs are represented in each cohort (not necessarily in all replicates within that cohort), it is counted as occurring there. We then used the neutral modelling approach to partition these core ASVs to those that are neutral, and those that are above/below the model fit (deterministically/assembled). To draw these ASVs, we have used the R's metacoder package (Foster, Z. S., Sharpton, T. J., & Grünwald, N. J. (2017). Metacoder: An R package for visualization and manipulation of community taxonomic diversity data. *PLoS computational biology*, *13*(2), e1005404).

Differential Analysis

To find ASVs that were significantly different between multiple categories considered in this study, we used the DESeq2 package (Love, Huber, and Anders 2014) with the adjusted p-value significance cut-off of 0.05 and log fold change cut-off of 2.0. This function uses negative binomial GLM fitting to obtain maximum likelihood estimates for the ASVs log fold change between the two conditions. Bayesian shrinkage was then applied to obtain shrunken log fold changes, subsequently employing the Wald test for obtaining significances.

CODA-LASSO

As opposed to GLLVM, which regresses individual feature abundance against all sources of variation, in CODA-LASSO we do the opposite. We take a single clinical covariate (a variable with continuous outcome) and try to find a minimal subset of features, a composition of

these, have a relationship with the covariate of interest. For this purpose, we employ log-contrast functions and composition balance approach (Susin et al., 2020) where the goal is to identify two disjoint subsets of microbes, those that are positively associated, and those that are negatively associated with the covariate of interest. This is done through the CODA-LASSO approach (Lu et al., 2018) where the abundance of individual covariate y_i is modelled as $y_i = \beta_0 + \beta_1 \log(x_{1i}) + \cdots + \beta_j \log(x_{ji}) + \epsilon_i$ (for i-th sample and j-th species, with x_{ji} being the microbe abundance) with the constraint $\sum_{k\geq 1} \beta_k = 0$ (i.e., all β-coefficients sum up to 0), and these regression coefficients $\boldsymbol{\beta} = (\beta_0, \ldots, \beta_j)$ are estimated to minimise $\sum_{i=1}^{n} (y_i - \beta_0 - \beta_1 \log(x_{1i}) - \cdots - \beta_j \log(x_{ji}))^2 + \lambda \sum_{k\geq 1} |\beta_k|$ subject to $\sum_{k\geq 1} \beta_k = 0$ (using a soft thresholding and projection algorithm) for n samples. Here, λ is the penalization parameter in Lasso shrinkage terms $\lambda \sum_{k\geq 1} |\beta_k|$ which forces some of the $\boldsymbol{\beta}$-coefficients to go to zero, particularly those that do not have a relationship with the covariates, and thus the non-zero $\boldsymbol{\beta}$-coefficients (which are associated with a subset of microbes) serves as a mean to enable variable selection. The non-zero $\boldsymbol{\beta}$-coefficients are then divided into two groups, those that are positively associated with the clinical covariate, and those that are negatively associated with the clinical covariate, respectively. The procedure is implemented as coda_glmnet() function in R's coda4microbiome package(Calle & Susin, 2022). Similar to GLLVM, we have used the top 100 most abundant genera, however, after filtering out for microbes with an occupancy threshold of 10 (i.e., genera that are non-zero in at least 10 samples).

Quantitative Cluster Association test (QCAT-C)

In medical datasets, the study design is such that a single subject provides multiple samples either over a course of treatment, or for different body regions. As a result, the data is paired, and this association has to be incorporated in a statistical test to reduce the impact of inherent correlations that exist as a result of this paired nature. For this purpose, we have used a specialised cluster association test (Tang & Chen, 2021) utilising R's miLineage package (https://tangzheng1.github.io/tanglab/software.html) with this test referred to as QCAT-C test using the QCAT_GEE.Cluster() function. The QCAT-C test is a two-part test where it fits separate models to microbes that are observed as excessively zero in the samples, and those that have predominantly positive abundances (referred to as positive microbes). Additionally, the test incorporates collating abundances up the taxanomic hierarchy to give differential abundance at different taxanomic levels simultaneously. To visualise the differentially abundant taxa at different taxonomic ranks, we have used Total Sum Scaling following by Centralized Log Ratio (TSS+CLR) transform on raw abundances.

Kendall Rank Correlation Analysis

Correlation analysis was performed using the Kendall rank correlation coefficient, and Bonferonni adjustment was used to generate the p values (BONFERRONI and C. 1936).

Bray Curtis Contributions

To see how a subset of features (microbes or a subset of KEGG orthologs (KOs)) vary within a cohort (i.e., all categories under which samples are organized), we calculated how much beta diversity is contributed either by the subset of features observed in the dataset. For this purpose, we employed the *Bray-Curtis* (BC) dissimilarity as a metric of community dissimilarity is defined as $BC_{jk} = \frac{\sum |X_{ij}-X_{ik}|}{\sum (X_{ij}-X_{ik})}$, where BC is the Bray-Curtis dissimilarity between communities j and k and X is the relative abundance of feature i. Since BC is a scaled summation of abundance differences between two communities, we can easily partition BC dissimilarity between two samples attributable to a subset of the features. To obtain the contribution of the subset of features, for two samples, we calculate the BC twice, once with calculating the summation in the numerator of the BC expression but use subset of features (BC_subset), and once with all the features (BC_all). There is no change in denominator of the

above function. Dividing BC_subset by BC_all then reports the fraction of beta diversity attributed to the subset of features. This is implemented in R's otuSummary package (Yang, 2018). To see if the Bray-Curtis contribution was significant between groups, we used Tukey Honest Significance Difference (HSD) test from R's Stats package. The scripts and workflows used to carry out all of the above bioinformatics and analyses can be found at http://userweb.eng.gla.ac.uk/umer.ijaz#bioinformatics.

Supplementary Figure 1| Taxa plots representing the top 25 most abundant ASVs (top panel) and genera (bottom panel) observed in all samples.

Supplementary Figure 2| QCAT association test. *Subset of taxa (at all levels) that are differentially abundant between the cohorts considered in this study using QCAT-C association test that considers paired nature of samples i.e., originating from the same subject connected by lines. The values represent the TSS+CLR (Total Sum Scaling followed by Centralised Log Ratio transform) normalized abundances of individual taxa. The procedure returns two measures of significances, a local p-value that is applied to every taxa (in this case all local p values are < 0.05 including taxa represented in the supplementary figures), and a single global p-value considering all the differentially abundant lineages together (in this case, global p < 0.001 for all lineages returned).*

Supplementary Figure 3| Null Modelling. *A combined null-model approach to identify and quantify ecological community assembly processes in CKD groups using* ***(a)*** *environmental filtering calculated as nearest taxa index (NTI) where values >+2 indicate extreme clustering in the phylogenetic tree driven by environmental pressures (determinism), where lines connecting two categories show significant differences (ANOVA) with * (p < 0.05), ** (p < 0.01), or *** (p < 0.001).* ***(b)*** *quantitative process estimates (QPE) approach which determines the proportion of assembly mechanisms acting on a category within the framework of selection, dispersal and drift represented by a stacked bar chart; the* ***(c)*** *the stochasticity ratio approach, which quantified stochasticity using the Jaccard metric and Taxa-Richness constraints of P-P and P-F in generating the null communities. We have used two measures, normalised stochasticity ratio (NST) and a modified stochasticity ratio (MST), as proposed by the authors of this method.*

Supplementary Figure 4a Nonzero *β*- coefficients returned from CODA-LASSO procedure as two disjoint sets (those that are positively related, and those that are negatively related with the calcium, eGFR, and TMAO) for only those samples that belong to KTx baseline. The predictions on the right from the fitted model through CODA-LASSO was further regressed against the true calcium, eGFR, and TMAO values and show agreement. For example, the R value for TMAO was high (0.78), and so this model showed a good fit.

Supplementary Figure 4b Nonzero *β*-coefficients returned from CODA-LASSO procedure as two disjoint sets (those that are positively related, and those that are negatively related with the age, hsCRP, calcium, choline, eGFR, albumin, cholesterol, betaine) for only those samples that belong to CKD 3-4. The predictions on the right from the fitted model through CODA-LASSO was then further regressed against the true age, hsCRP, calcium, choline, eGFR, albumin, cholesterol, betaine TMAO values and show agreement.

Supplementary Figure 4c Nonzero *β*- coefficients returned from CODA-LASSO procedure as two disjoint sets (those that are positively related, and those that are negatively related with the IL-6, calcium, albumin, choline, betaine) for only those samples that belong to CKD5. The predictions on the right from the fitted model through CODA-LASSO was further regressed against the true IL-6, calcium, albumin, choline, betaine values and show agreement.

Supplementary Figure 5| Bray-Curtis Contributions. Bray-Curtis contribution of genes to the overall beta diversity within the categories. The plot shows *N(N-1)/2* pair-wise differences between samples for each category (CKD3-4, CKD5, KTx baseline, KTx one year follow up) with the left set of panels showing beta diversity contribution by all KOs that are not in the list of KOs associated with the chosen pathway, whilst the right set of panels showing organization by, Choline metabolism in cancer, Chemical Carcinogenesis-Reactive oxygen species, Betaine biosynthesis, Longevity related pathway, and AMR respectively. Higher contributions represent higher inter-sample variability in terms of pathway-associated genes.
